# Why do you think you should be the author on this manuscript? Analysis of open-ended responses of authors in a general medical journal

**DOI:** 10.1186/1471-2288-12-189

**Published:** 2012-12-20

**Authors:** Mario Malički, Ana Jerončić, Matko Marušić, Ana Marušić

**Affiliations:** 1 Department of Research in Biomedicine and Health, University of Split, School of Medicine, Split, Croatia

**Keywords:** Authorship, Guideline adherence, Contribution disclosure form, International Committee of Medical Journal Editors (ICMJE), Editorial policies, Croatia

## Abstract

**Background:**

To assess how authors would describe their contribution to the submitted manuscript without reference to or requirement to satisfy authorship criteria of the International Committee of Medical Journal Editors (ICMJE), we analyzed responses of authors to an open-ended question *“Why do you think you should be the author on this manuscript?”*.

**Methods:**

Responses of authors (n=1425) who submitted their manuscripts (n=345) to the *Croatian Medical Journal*, an international general medical journal, from March 2009 until July 2010 were transcribed and matched to ICMJE criteria. Statements that could not be matched were separately categorized. Responses according to the number of authors or their byline position on the manuscript were analyzed using Mann–Whitney *U* test and Moses test of extreme reactions.

**Results:**

The number of authors per manuscript ranged from 1 to 26 (median=4, IQR=3-6), with the median of 2 contributions per author (IQR=2-3). Authors’ responses could be matched to the ICMJE criteria in 1116 (87.0%) cases. Among these, only 15.6% clearly declared contributions from all 3 ICMJE criteria; however, if signing of the authorship form was taken as the fulfillment of the third ICMJE criterion, overall fraction of deserving authorship was 54.2%. Non-ICMJE contributions were declared by 98 (7.6%) authors whose other contributions could be matched to ICMJE criteria, and by 116 (13.0%) authors whose contributions could not be matched to ICMJE criteria. The most frequently reported non-ICMJE contribution was literature review. Authors on manuscripts with more than 8 authors declared more contributions than those on manuscript with 8 or fewer authors: median 2, IQR 1–4, vs. median 2, IQR 1–3, respectively (Mann Whitney *U* test, p=0.001; Moses Test of Extreme Reactions, p<0.001). Almost a third of single authors (n=9; 31.0%) reported contributions that could not be matched to any ICMJE criterion.

**Conclusions:**

In cases of multi-author collaborative efforts but not in manuscripts with fewer authors open-ended authorship declaration without instructions on ICMJE criteria elicited responses from authors that were similar to responses when ICMJE criteria were explicitly required. Current authorship criteria and the practice of contribution declaration should be revised in order to capture deserving authorship in biomedical research.

## Background

Authorship is perhaps the most important aspect of research – it recognizes the credit for research and, what is important for individual scientists, it is the primary criterion for career advancement. Although it may seem that giving credit for research would be a straightforward decision, authorship is burdened by many problems in all research disciplines [1]. In biomedicine, there is ample evidence that the authorship criteria widely accepted by journals and publishers – those from the International Committee of Medical Journal Editors (ICMJE) [2] are not well understood or followed by both the medical students and experienced researchers [3-5], resulting in a high prevalence of authors of published articles who do not satisfy the ICMJE criteria [6-9].

Our research group has shown in a number of studies in our own and other journals that forms used by journals for contribution declaration for authorship are not a reliable way of judging authorship [8-12], and that authorship does not seem to be only a normative issue subjective to categorization into criteria, but also a very personal view of the importance and value of one's contributions [13]. Based on this body of evidence, we hypothesized that authors who were asked to describe in their own words why they think they deserve authorship on a submitted manuscript may differ in their contribution declaration then the authors who were instructed about standard authorship criteria or asked to declare authorship using checklists with ICMJE-eligible contributions.

## Methods

### Participants

All authors (n=1425) who submitted manuscripts (n=345) to the *Croatian Medical Journal* (CMJ) from March 2009 until July 2010 were included in the study. Individual forms with a question about authorship and other information (contact address, copyright and participation in editorial research) for each author of the manuscript were sent by e-mail to the corresponding authors, who were asked to distribute these to their coauthors. Completed and signed documents were returned to the editorial office by the individual or corresponding authors.

### Ethical considerations

The participation in the study was voluntary and did not influence the editor's decision to accept or reject the articles submitted. As the full information on the study could influence the response of the authors, authors were asked to accept the participation in research of editorial and peer-review issues [9-12]. This information was provided in the authorship form and the authors were offered an opt-out option for the participation in the journal's research. The study was approved by the Ethics Committee of the Zagreb University School of Medicine under the grant from the Ministry of Science and Technology of the Republic of Croatia No. 108-1080314-0140 to MMar.

### Authorship form

The form started with the general definition of an author: “An AUTHOR of a scientific article is considered to be someone who has made substantive contribution to the submitted work.” There was no reference to any authorship criteria. We then asked the authors about their contribution to the submitted work in the following way: “The CMJ requests from the authors of submitted manuscripts to describe their contribution to the research described in the manuscript by answering the following question: Why do you think you should be the author on this manuscript?”

### Analysis of responses

All responses were transcribed into an Excel spreadsheet, including the authors byline position in the submitted manuscript, and the manuscripts submission number. Wording the ICMJE used for defining authorship credit formed the basis of variables (criteria) to which the authors’ statements were manually matched [2]: “Authorship credit should be based on 1) substantial contributions to conception and design, acquisition of data, or analysis and interpretation of data; 2) drafting the article or revising it critically for important intellectual content; and 3) final approval of the version to be published. Authors should meet conditions 1, 2, and 3.” Authorship statements that could not be matched to any of the above ICMJE criteria, such as translation of the article, supervision/mentorship or literature search, were separately categorized.

### Statistical analysis

Frequencies and percentages were used for the description of qualitative variables. Depending on the distribution of data, following descriptors were used for quantitative variables: mean and standard deviation, median and interquartile range (IQR). Pearson's chi-square test was used to compare qualitative data frequencies. The distributions of total authors on paper according to identical responses by authors were contrasted using the Mann–Whitney *U* test and the Moses test of extreme reactions, to analyze differences in central tendency and dispersion, respectively. The same two tests were used to compare the number of declared contributions per author by total author count or by author’s byline. A 95% confidence interval (CI) for median, estimated by the bias-corrected and accelerated bootstrapping method with 2000 replications, was also reported in order to complement hypothesis testing. The level of significance for all statistical tests was 0.05. Data were analyzed with SPSS statistical package 19.0 (SPSS; Chicago, Illinois, USA).

## Results

We analyzed responses from 1282 (90.0%) authors of 335 submitted manuscripts; 140 (9.8%) authors signed their acceptance to participate in research but did not write an answer to the authorship question; 1 form was not readable, and 2 referred to attachments which were not available for analysis. The number of authors per article ranged from 1 to 26 (median=4, IQR=3-6). The median number of declared contributions per author was 2 (IQR=2-3).

Authors’ responses could be matched to ICMJE criteria in 1116 (87.0%) cases (Table 1). Among these authors, only 15.6% clearly declared contributions from all three ICMJE categories, whereas 3 authors (0.3%) explicitly stated that they satisfied ICMJE criteria. More than a third (38.6%) satisfied the first two ICMJE criteria (research and writing), while the rest declared a single ICMJE contribution, either to research execution (31.8%) or writing (9.4%) (Table 1). Authors whose contributions could be matched to the ICMJE criteria used the wording that was similar to the terminology used in the ICMJE definition (Table 2). The frequency of the terminology identical to that from the ICMJE definition ranged from 52.8% for “data collection” to 98.0% for “study design”. Other declarations used words or phrases that described the activity covered by the ICMJE criteria.

**Table 1:** Number (%) of authors (n=1116) whose authorship statement could be matched to ICMJE criteria

**Criteria**	No. (%) of authors*
Full criteria (1 and 2 and 3)	174 (15.6)
Criteria 1 and 2 not 3	431 (38.6)
Criteria 1 and 3 not 2	26 (2.3)
Criteria 2 and 3 not 1	17 (1.5)
Criterion 1 only:	355 (31.8)
“conception”	12 (3.4)
“design”	13 (3.7)
“acquisition of data”	161 (45.3)
“analysis of data”	24 (6.8)
“interpretation of data”	9 (2.5)
any 2 contributions	92 (25.9)
any 3 contributions	34 (9.6)
any 4 contributions	9 (2.5)
all 5 contributions	1 (0.3)
Criterion 2 only:	105 (9.4)
“drafting of the article”	45 (42.8)
“revising of the article”	49 (46.7)
both contributions	11 (10.5)
Criterion 3 only	5 (0.5)
Statement: “Because I contributed to the submitted article as an author according to the ICMJE criteria”	3 (0.3)

*Percentages for contributions specified in an individual authorship criterion are to the total sum of responses within that criterion.

**Table 2:** Expressions used by authors (n=1116) in statements that could be matched to the ICMJE criteria

**Expression categories**	**No. (%) of authors**
*Criterion 1 – Conception:*	
“planned/planning”	129 (43.7)
“conception”	118 (40.0)
“my idea“	32 (10.1)
“organized research”	5 (2.0)
“hypothesis”	5 (2.0)
Other*	6 (2.2)
*Criterion 1 – Design:*	
“design/designing”	347 (98.0)
“chose proper methodology”	3 (0.9)
“experiment construction”	2 (0.5)
Other†	3 (0.6)
*Criterion 1 – Acquisition of data:*	
“data collection”	183 (31.1)
“data acquisition”	128 (21.7)
“did the experiments”	77 (13.1)
“conducted the study”	52 (8.8)
“treatment”	32 (5.4)
“diagnosis”	30 (5.1)
Other‡	87 (14.8)
*Criterion 1 – Analysis of data:*	
“analysis made/analyzed”	257 (73.7)
“statistical analysis”	87 (24.9)
“processed data”	5 (1.4)
*Criterion 1 – Interpretation of data:*	
“interpretation”	255 (91.8)
“presentation”	19 (6.8)
“draw conclusion”	3 (1.4)
*Criterion 2 – Drafting of the article:*	
“writing”	247 (46.9)
“drafting”	182 (34.5)
“manuscript preparation”	84 (15.8)
“wrote discussion”	7 (1.4)
Other§	7 (1.4)
*Criterion 2 – Revising the article:*	
“revision/revising”	219 (73.5)
“intellectual contribution”	54 (18.1)
“edited”	18 (6.0)
“made final copy/version”	5 (1.6)
“made corrections”	3 (1.0)
Other ∥	5 (1.6)
*Criterion 3 – Final approval:*	
“final approval”	215 (91.8)
“agree with the version published”	5 (2.1)

*Statements containing terms such as “inception”; “initiation”; “developed the project”.

†Statements containing terms such as “depicted the original scheme”; “protocol construction”.

‡Statements containing terms such as “obtained data”; “gathered data”; “did research”; “patient management”.

§Statements containing terms such as “wrote introduction”; “wrote results”; “wrote abstract”.

∥Statements containing terms such as “second draft”; ”completion”; “polishing”.

The responses of 166 (13.0%) authors could not be matched to ICMJE criteria (Table 3). Among them 56.3% stated that they made a significant contribution, without listing what that contribution was, while others declared the importance of the submitted case, employment in a health care institution, or just wrote “Yes”. Some also reported mentorship/supervision, literature search and translation, administrative/technical/statistical support and other professional contributions as valid reasons for authorship. Three authors stated that the submitted work was a part of their master thesis – they were the first authors on manuscripts with 3 (2 manuscripts) and 6 authors. Non-ICMJE contributions were also declared by 98 (8.8%) authors whose other contributions could be matched to ICMJE criteria (Additional file 1: Table S1). Among these authors, additional contributions were declared most often by those who also declared contributions to the first two ICMJE criteria (50.0%), while the most frequently reported contribution was literature review, in 52.0% cases.

**Table 3:** Number (%) of authors (n=166) whose authorship statement could not be matched to ICMJE criteria for authorship

**Stated contribution**	No. (%) of authors*
Substantial/direct contribution	94 (56.6)
“Yes”	9 (5.4)
This (case) is interesting	8 (4.8)
We work/collaborate together	8 (4.8)
I am a specialist of/I work in the department for	7 (4.2)
“I am interested in this topic/field”	6 (3.6)
“It was my master degree thesis”	3 (1.8)
“The study carries scientific value”	3 (1.8)
Literature search	3 (1.8)
“I have participated sufficiently to take public responsibility for appropriate portions of the content”	3 (1.8)
Chief of the project/department	3 (1.8)
Supervisor	2 (1.2)
Coordinator	2 (1.2)
Bibliographical search, administrative and logistic support	2 (1.2)
“I have other related publications as a biostatistician“	2 (1.2)
Signature of the author	2 (1.2)
“My participation in the contribution was administrative and technical support.”	1 (0.6)
“I was responsible for translation of the article.”	1 (0.6)
“I have especially taken part in collecting the literature data, translation.”	1 (0.6)
“This is the first study of its kind.”	1 (0.6)
“On the basis of operational practice and clinical experience which led to this work.”	1 (0.6)
“Because I want to present process of treatment in our institute.”	1 (0.6)
“I am an investigator in scientific project with similar problems.”	1 (0.6)
“Because I believe this is a proper treatment in this situation.”	1 (0.6)
“I was scientific consultant of this research”	1 (0.6)

*Percentages do not add up to 100 because of rounding.

For all respondents, open-ended declarations were mostly in the form of a full or partial sentence: 655 authors (55.1%) used the sentence starting with “I …”, 108 (8.4%) authors started with “Because …”, and 15 (1.2%) with “My …”. The rest of the respondents (n=401, 31.3%) just listed their contributions (Additional file 2: Table S2).

**Figure 1:**
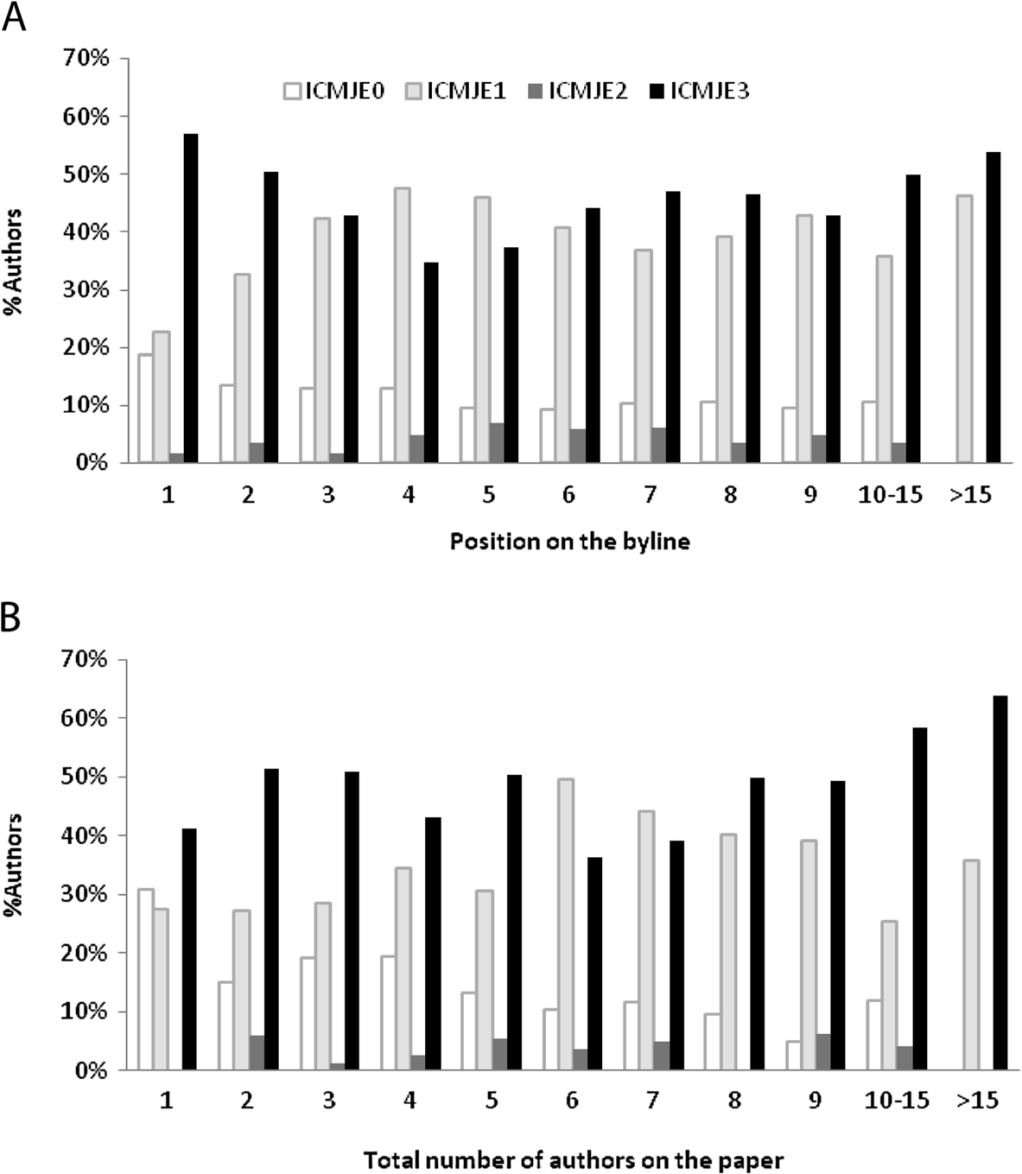
**Distribution of authors’ declared contributions according to their position on the byline (A) and the total number of authors on a manuscript (B).** ICMJE 0 – authors whose contributions could not be matched to ICMJE criteria, ICMJE 1, 2 or 3 – authors whose contributions could be matched to one, two or all three ICMJE criteria, respectively.

We separately analyzed the distribution of authors with ICMJE matching and non-matching contributions in relation to their position on the byline and the total number of authors on the submitted manuscript. Single authors on a manuscript (n=29) most often reported contributions that satisfied all 3 ICMJE criteria (n=12; 41.4%), while 8 (27.6%) of them reported a single ICMJE contribution. Almost a third of single authors (n=9; 31.0%) reported contribution(s) that could not be matched to any ICMJE criterion. The reasons for authorship they declared could be grouped into 2 distinctive sets: a) significant contribution to the reported work, without specification of contribution(s) and b) professional competence in the field from which the research was reported. Similar declarations were observed for manuscripts with 3 authors (31 out of 161 authors, 19.3%, declared non-ICMJE matching contributions). In manuscripts with 2 authors, authors whose contributions could not be matched to the ICMJE criteria (10 out of 66, 15.2%) reported significant (unspecified) contributions. The fraction of authors with ICMJE non-matching contributions decreased with more authors on a manuscript, as well as with the ascending position on the byline (Figure 1). Authors with all 3 ICMJE criteria fulfilled (considering their signature as the fulfillment of the third criterion) predominated in all manuscripts, regardless of the number of authors or of their position on the byline, except for authors on the 3rd to the 5th byline position, who more often reported a single ICMJE matching contribution (Figure 1). In relation to their byline position, there was no difference between ICMJE matching and non-matching contributions declared on manuscripts with 2 or 3 authors (p>0.05 for all comparisons, Pearson’s chi-square). We observed a difference in the number of contributions declared by ICMJE matching and non-matching statements. In general, authors on manuscripts with more than 8 authors declared more contributions than those on manuscript with 8 or fewer authors: median 2, IQR 1–4 (95% CI for median 2–3), vs. median 2, IQR 1–3 (95% CI 2–2), respectively (Mann Whitney *U* test, p=0.001; Moses Test of Extreme Reactions, p<0.001). This difference was still observed in the subsequent analysis when we compared the number of ICMJE matching contributions by authors between manuscripts with more than 8 authors (median 3, IQR 2–4, 95% CI 2–3), and less or equal to 8 authors (median 2, IQR 1–4, 95% CI 2–2) (Mann Whitney *U* test, p=0.061; Moses Test of Extreme Reactions, p<0.001). The difference in the number of contributions in manuscripts authored by researchers whose contributions could not be matched to the ICMJE criteria was inconclusive as the analysis was underpowered (only 17 out of 166 authors (10.2%) were authors on manuscripts with more than 8 authors). The position of those authors on the byline was not associated with the number of contribution declarations. Those with the byline position higher than 8^th^ had a median of 2 contributions, IQR 1–3 (95% CI 2–2), similar to authors with lesser or equal to the 8^th^ byline position which also contributed a median of 2 contributions, IQR 1–3 (95% CI 1.5–3) (Mann Whitney *U* test, p=0.762; Moses Test of Extreme Reactions, p=0.065).

There were 95 manuscripts where contribution declarations had identical wording for 2 or more authors. Such manuscripts tended to have a greater total number of authors than the whole population of manuscripts (Additional file 3: Figure S1): whereas all manuscripts had median of 4 authors (IQR=4-6), manuscripts with identical declarations had a median of 6 authors (IQR=4-8). Significant differences were found both in the central tendency (Mann Whitney *U* test, p<0.001) and the dispersion (Moses Test of Extreme Reactions, p<0.001) between the two groups of manuscripts.

## Discussion

Our study showed that authors, when asked about authorship in a non-instructional way, i.e. without reference to the ICMJE criteria as a standard in biomedicine [2], mostly declared contributions that could be matched to the first two ICMJE criteria (executing research and writing the manuscript), but not to the third ICMJE criterion (approving of the final manuscript version). The authors also most often used the wording of the ICMJE definition to describe their contributions. The declarations from 13% of the authors could not be matched to any ICMJE criterion. The fraction of such authors decreased with the increasing number of authors and their remoteness from the first byline position.

Our study is limited by its cross-sectional design, but our findings are strengthened by a high response rate, since filling out authorship forms is mandatory for manuscript processing in most science journals. Generalizability of the findings to other medical journals and research and academic settings can also be questioned as the study was performed in a single journal. However, the results are consistent with the previous finding from authorship studies in our journal, which included both survey and randomized study designs [9-12], as well as with studies from other journals or academic settings [3,4,6-8].

Despite the fact that we asked an open-ended question and did not provide instruction on ICMJE definition as accepted authorship criteria in biomedicine, most of the reported contributions matched those described in the ICMJE definition of authorship. However, only 15.6% of the authors whose contributions could be matched to ICMJE definition satisfied all three ICMJE criteria. Further 38.6% declared contributions exclusively to the first two ICMJE criteria (research and writing). As authors made this declaration on a signed statement after manuscript submission, it can be assumed that they gave approval to the manuscript submitted to the journal. If their signature is then taken as a fulfillment of the third ICMJE criterion, the overall fraction of deserving authorship according to the ICMJE increases to 54.2%, which is similar to the results of our previous studies (range from 39% to 75%) that were based on the ICMJE definition and which had different study designs [9-12]. Approval of the manuscript can be regarded as something that is outside of the creative effort of researchers, and it can even be impossible to obtain in cases when one of the researches dies before the final version of the manuscript is finished. Today many journals require contact e-mails from all of the listed authors, subsequently informing them that the corresponding author had submitted an article in their name. Consequently, this makes the final approval a procedural requirement, and not necessarily a criterion for authorship contribution.

The lack of regard for final approval as a criterion for deserved authorship observed in this study, which had a cross-sectional design and did not refer to the ICMJE criteria, confirms the results of our previous study where we showed in a randomized study design that the “final approval of the article” was an inherently different category from other contributions and that it should be considered rather as an administrative requirement similar to signing of a copyright transfer [11]. Furthermore, our recent analysis of journals from different research fields, including social sciences and humanities, also demonstrated that authorship definitions by journals, publisher and professional organizations or associations mostly addressed research and/or writing as contributions necessary for authorship [14]. In our study, authors who declared a single contribution that could be matched to an ICMJE criterion, declared research contribution more often (in 31.8% cases) than writing contribution (in 9.4% cases).

The “fractionation” of authorship into more contribution categories was evident in multi-authored articles, where the number of contributions declared increased in manuscripts with more than 8 authors and specifically in those where authors declared ICMJE matching contributions. Taken together with the finding that overall, non-ICMJE matching contributions were more frequent in manuscripts with few authors (1–3 per byline), this indicates that authors from smaller research collaborations do not see the need to elaborate on their contributions, as their role in research presented is clear. In larger collaborative groups, contribution declaration seems to require a coordinated effort, particularization and careful distribution of contributions. Our study was not designed to check whether each individual author really filled in the form (although each was separately signed by individual authors), but the finding that manuscripts with more than 6 authors had more declaration forms with identical wording of the declarations indicates that filling in the forms could either be a centralized effort to formally satisfy journal’s requirements or that authors simply copied from each other, without engaging in truthful elaboration of their contributions. Different behavior in authorship declaration with increasing number of authors on manuscripts can also be related to the current publishing practices in biomedicine, where the number of authors was not perceived as an important issue for academic performance, in contrast to the position on the byline and the journal’s impact factor, explaining at least in part the increase in the number of authors per publication [15].

Single authors or authors of manuscripts with 2 to 4 authors had most ICMJE non-matching contribution statements. These authors usually stated that they made a significant contribution, without any specification, or they disclosed their professional expertise in the field. These statements, however, cannot be taken to imply undeserved authorship; they rather suggest differences in perceptions of the authors to the established criteria for authorship and the means of their reporting.

Despite the fact that our authorship forms had a required line where the authors had to sign their name if they agreed to be listed as an author in the submitted manuscript; a small number of authors, who answered our authorship question with only a “Yes”, most likely perceived that question as just another required confirmation of their authorship. Perception of authorship declarations as a form of external check-list necessary for manuscript submission is also supported by the finding that almost a third of the respondents in our study did not use full or partial sentences to a question that required an answer in the form of a sentence. Providing a list of contributions instead of a sentence may be the result of authors’ experience and familiarity with the prevalent practice in biomedical journals to formulate their contribution declarations as checklists.

## Conclusion

Based on our previous research into authorship [9-12], particularly our finding that authorship is not a normative issue subjective to categorization into criteria, but a very personal view of the importance and value of one's contributions [13], we hypothesized that journals should ask the authors a simple questions “Why do you think you deserve to be the author of this manuscript?” [9]. The current study demonstrated that such an open-ended authorship declaration without instructions on any available authorship criteria elicits responses from authors that are similar to responses when the ICMJE criteria are explicitly required. Contribution declaration is especially problematic in multi-authored collaborative research efforts. Taken together with the results of our recent systematic review on authorship research [1], particularly the disappointing findings that the practice of contribution declaration has not reduced the number of authors on the byline [16,17], there is enough evidence that current authorship criteria in medicine are not adequate and that they should be revised to capture those deserving authorship in biomedical research. We believe that obligatory inclusion of authorship issues in research education, as well as planning of authorship during the development of research protocols would enable fair recognition of all contributions to the research effort. The task of journal editors in this system would not be that of regulating authorship criteria and monitoring authorship eligibility, but that of maintaining public trust in the research enterprise by ensuring the transparency of the authorship decision process.

## Competing interests

At the time of the study, A. Marušić and M. Marušić were Coeditors in Chief of the *Croatian Medical Journal*. The authors declare that they have no competing interest.

## Authors’ contributions

MMal – Created the database, matched authors statements to ICMJE criteria, performed parts of descriptive statistics, interpreted results, drafted the abstract and tables, revised the initial draft of manuscript, and approved the final version of the manuscript. AJ – Helped create the database, performed statistical analysis, interpreted results, drafted the methods section and prepared figures, revised the initial draft of manuscript, and approved the final version of the manuscript. MMar – Conceived and designed the study, acquired data, coordinated coauthors, interpreted results, revised the initial draft of manuscript, and approved the final version of the manuscript. AM – Conceived and designed the study, reviewed matching of authors statements to ICMJE criteria, performed literature review, interpreted results, drafted the initial manuscript, revised the coauthors inputs and additions, and approved the final version.

## Pre-publication history

The pre-publication history for this paper can be accessed here:

http://www.biomedcentral.com/1471-2288/12/189/prepub

## Supplementary Material

Additional file 1 **Table S1.** Contribution declarations of authors (n=98) whose free text authorship statements included both ICMJE matching and non-matching contributions. Click here for file

Additional file 2 **Table S2.** Sentence structure of authors’ answers (n=1282) to the open-ended question: Why do you think you should be the author on this manuscript? Click here for file

Additional file 3 **Figure S1.** Distribution of manuscripts with (closed bars) or without (open bars) identical contribution declarations from at least 2 authors according to the number of authors on the manuscript. Click here for file
