# Supplementary material for: Why do you think you should be the author on this manuscript? Analysis of open-ended responses of authors in a general medical journal
Source: BMC Med Res Methodol. 2012 Dec 20;12:189. doi: 10.1186/1471-2288-12-189 (PMC3552823; doi:10.1186/1471-2288-12-189)
Supplement: Additional file 1 — Table S1. Contribution declarations of authors (n=98) whose free text authorship statements included both ICMJE matching and non-matching contributions. [file 1471-2288-12-189-S1.doc]

**Table S1.** Contribution declarations of authors (n=98) whose free text authorship statements included both ICMJE matching and non-matching contributions

| **ICMJE non-matching contribution** | **No. of authors declaring contributions to ICMJE criterion** | | | | |
| --- | --- | --- | --- | --- | --- |
|  | **1** | **2** | **1+2** | **2+3** | **1+2+3** |
| Literature review | 14 | 7 | 27 | 0 | 3 |
| “I organized the sponsorship.” | 0 | 0 | 0 | 0 | 1 |
| “Soliciting coauthors for productive discussion” | 0 | 0 | 0 | 0 | 1 |
| Technical support | 3 | 0 | 0 | 0 | 1 |
| I am a specialist of/I work in the department for | 3 | 3 | 2 | 0 | 0 |
| Willingness to guarantee the integrity of the entire study | 0 | 0 | 5 | 0 | 0 |
| Obtained funding | 0 | 0 | 2 | 0 | 0 |
| Supervisor | 0 | 1 | 2 | 0 | 0 |
| Consultant of the residency thesis | 2 | 0 | 0 | 1 | 0 |
| Chief of the project/department | 0 | 0 | 1 | 0 | 0 |
| Translation | 0 | 2 | 1 | 0 | 0 |
| Thesis mentor | 1 | 1 | 1 | 0 | 0 |
| Worked on the publication plan | 0 | 0 | 1 | 0 | 0 |
| Thesis | 2 | 0 | 1 | 0 | 0 |
| Because this is an original study | 0 | 0 | 1 | 0 | 0 |
| Coordinator | 0 | 2 | 1 | 0 | 0 |
| Literature review and obtained comments from coauthors | 0 | 0 | 1 | 0 | 0 |
| Literature review and funds collection | 0 | 0 | 1 | 0 | 0 |
| Study supervision and fund collection | 0 | 0 | 1 | 0 | 0 |
| I am interested in the field. I learned a lot during writing of this manuscript, which will result in the better treatment of this disease. | 0 | 0 | 1 | 0 | 0 |
| Literature review and interested in the field | 1 | 0 | 0 | 0 | 0 |
| Total | 26 (26.6%) | 16 (16.3%) | 49 (50.0%) | 1 (1.0%) | 6 (6.1%) |

*ICMJE criteria: 1 – substantial contributions to conception and design, acquisition of data, or analysis and interpretation of data, 2 – drafting the article or revising it critically for important intellectual content, 3 – final approval of the version to be published, 1+2, 2+3, 1+2+3 – combinations of ICMJE individual criteria
