# Supplementary material for: Why do you think you should be the author on this manuscript? Analysis of open-ended responses of authors in a general medical journal
Source: BMC Med Res Methodol. 2012 Dec 20;12:189. doi: 10.1186/1471-2288-12-189 (PMC3552823; doi:10.1186/1471-2288-12-189)
Supplement: Additional file 2 — Table S2. Sentence structure of authors’ answers (n=1282) to the open-ended question: Why do you think you should be the author on this manuscript? [file 1471-2288-12-189-S2.doc]

**Table S2.** Sentence structure of authors’ answers (n=1282) to the open-ended question: Why do you think you should be the author on this manuscript?

| **Sentence structure** | **No. (%) of authors*** |
| --- | --- |
| Simple sentence: subject + verb: | 869 (67.8) |
| beginning with “I” + verb | 655 (75.4) |
| beginning with “Because” | 108 (12.4) |
| beginning with “Due to” | 8 (0.9) |
| beginning with “For” | 3 (0.3) |
| beginning with “Thanks to” | 2 (0.2) |
| beginning with “Since” | 2 (0.2) |
| beginning with “On the basis of” | 1 (0.1) |
| beginning with “My participation/contribution” + predicate (verb) | 15 (1.7) |
| other (e.g. “This is the first study of its kind.”) | 75 (8.6) |
| Enumeration – listing of the contributions | 401 (31.3) |
| simple listing (e.g. “Acquisition of data, analysis of data.”) | 294 (73.3) |
| structured listing (participated/involved in, or contributed to) | 94 (23.4) |
| number style listing (e.g. 1)... , 2)… 3)… ) | 10 (2.5) |
| preceded by a colon (e.g. “Contribution to this article was:”) | 2 (0.5) |
| use of mathematical symbols (e.g. “Data generated in my lab + active participation in writing of manuscript.”) | 1 (0.2) |
| Pro-sentence (“Yes”) | 8 (0.6) |
| “Yes, + subject + verb | 4 (0.3) |

*****Percentages do not add up to 100 because of rounding. Percentages for subcategories are to the total sum of responses for the category.
