# Supplementary material for: Why do you think you should be the author on this manuscript? Analysis of open-ended responses of authors in a general medical journal
Source: BMC Med Res Methodol. 2012 Dec 20;12:189. doi: 10.1186/1471-2288-12-189 (PMC3552823; doi:10.1186/1471-2288-12-189)

**Figure 1.** Distribution of manuscripts with (closed bars) or without (open bars) identical contribution declarations from at least 2 authors according to the number of authors on the manuscript.


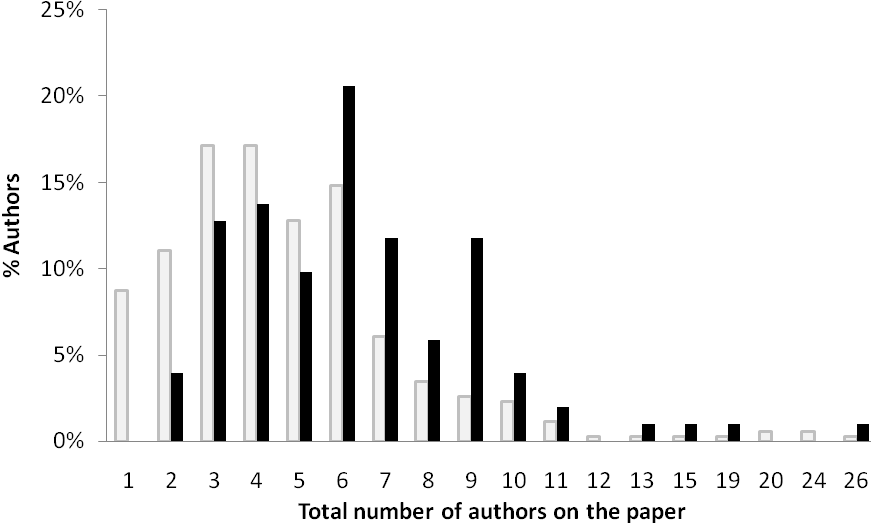

Supplement: Additional file 3 — Figure S1. Distribution of manuscripts with (closed bars) or without (open bars) identical contribution declarations from at least 2 authors according to the number of authors on the manuscript. [file 1471-2288-12-189-S3.doc]
